# 

**Published:** 1941

**Authors:** 


### CONTENTS.

| | Page |
|---|---|
| Medicine from Ancient Greece to Soviet Russia. By Professor B. Farrington, M.A. | 45 |
| Experiences of First Aid in Air Raids | 57 |
| The Casualty Surgery of Air Raids | 74 |
| Reviews of Books | 91 |
| Editorial Notes | 95 |
| Obituary: H. Elwin Harris, M.A., M.B., F.R.C.S. | 97 |
| The Medical Library of the University of Bristol | 98 |
| Meetings of Societies | 101 |
| Publications Received | 101 |
| Local Medical Notes | 102 |

